# Mental health disorders research in the countries of the Organisation of Islamic Cooperation (OIC), 2008–17, and the disease burden: Bibliometric study

**DOI:** 10.1371/journal.pone.0250414

**Published:** 2021-04-23

**Authors:** Grant Lewison, Richard Sullivan, Cengiz Kiliç

**Affiliations:** 1 Department of Cancer and Pharmaceutical Sciences, Institute of Cancer Policy, Guy’s Hospital, King’s College London, London, United Kingdom; 2 Department of Psychiatry, and Stess Assessment and Research Centre (STAR), Hacettepe University, Ankara, Turkey; Universita degli Studi di Milano-Bicocca, ITALY

## Abstract

The 57 countries of the Organisation of Islamic Cooperation are suffering from an increasing burden from mental health disorders. We investigated their research outputs during 2008–17 in the Web of Science in order to compare them with the burden from different mental health disorders and in different countries. The papers were identified with a complex filter based on title words and journals. Their addresses were parsed to give fractional country counts, show international collaboration, and also reveal country concentration on individual disorders and types of research. We found 17,920 papers in the decade, with output quadrupling. Foreign contributions accounted for 15% of addresses; they were from Europe (7%), Canada + USA (5%) and elsewhere (3%). They were much greater for Qatar and Uganda (> 60%), but less than 10% for Iran and Turkey. Schizophrenia and bipolar disorder were over-researched, but suicide and self-harm were seriously neglected, relative to their mental health disorder burdens. Although OIC research has been expanding rapidly, some countries have published little on this subject, perhaps because of stigma. Turkey collaborates relatively little internationally and as a result its papers received few citations. Among the large OIC countries, it has almost the highest relative mental health disorders burden, which is also growing rapidly.

## 1 Introduction

### 1.1 Objective

The purpose of this paper is to compare the burden of mental illness in the 57 countries of the Organisation of Islamic Coöperation (OIC) with the amount and distribution of their research to see if their portfolios are appropriate for the mental health disorder challenges that they face. The countries are listed in Table 1 with their International Standards Organization (ISO) digraph codes. Most of them are in the Middle East or North Africa (MENA) region. They vary greatly in size, from the wealthiest in terms of Gross Domestic Product (GDP): Indonesia (USD 862 billion in 2015), Turkey (USD 718 bn) and Saudi Arabia (USD 646 bn), down to Comoros and Gambia, with GDP < USD 1 bn. We also sought possible changes that they could make that would enable them better to rise to these challenges. In particular, we investigated the role of international collaboration in helping them to improve their research capability.

**Table 1:** List of the 57 countries in the Organisation of Islamic Co-operation group, with their ISO2 codes.

*Countries*	*ISO2*	*Countries*	*ISO2*	*Countries*	*ISO2*
Afghanistan	*AF*	Guyana	*GY*	Pakistan	*PK*
Albania	*AL*	Indonesia	*ID*	Palestine	*PS*
Algeria	*DZ*	Iran	*IR*	Qatar	*QA*
Azerbaijan	*AZ*	Iraq	*IQ*	Saudi Arabia	*SA*
Bahrain	*BH*	Jordan	*JO*	Senegal	*SN*
Bangladesh	*BD*	Kazakhstan	*KZ*	Sierra Leone	*SL*
Benin	*BJ*	Kuwait	*KW*	Somalia	*SO*
Brunei	*BN*	Kyrgyzstan	*KG*	Sudan	*SD*
Burkina Faso	*BF*	Lebanon	*LB*	Surinam	*SR*
Cameroon	*CM*	Libya	*LY*	Syria	*SY*
Chad	*TC*	Malaysia	*MY*	Tajikistan	*TJ*
Comoros	*KM*	Maldives	*MV*	Togo	*TG*
Cote d’Ivoire	*CI*	Mali	*ML*	Tunisia	*TN*
Djibouti	*DJ*	Mauritania	*MR*	Turkey	*TR*
Egypt	*EG*	Morocco	*MA*	Turkmenistan	*TM*
Gabon	*GA*	Mozambique	*MZ*	U Arab Emirates	*AE*
Gambia	*GM*	Niger	*NE*	Uganda	*UG*
Guinea	*GN*	Nigeria	*NG*	Uzbekistan	*UZ*
Guinea Bissau	*GB*	Oman	*OM*	Yemen	*YE*

Note: Western Sahara is also an OIC country, although its status is not settled, and it carried out no research on mental health disorders.

### 1.2 Previous studies

There is an extensive literature on the bibliometrics of mental health disorders research. We found over 130 papers, of which more than 80 appeared since 2010. Most of them were about particular disorders, notably depression (19), suicide & self-harm (13), Alzheimer’s disease (11), and addiction and anxiety (10 each). We could find no previous bibliometric study of mental health in this particular political grouping, but mental health research in the 22 Arab countries in 2009–18 was investigated recently [1]. They found that the leading countries were Egypt, Saudi Arabia, and Lebanon, and that Arab output had expanded more rapidly than world production. Many of the OIC countries are in the lower middle and low income groups, and their mental health research outputs were examined by Large et al. [2]. They also found a rapid increase in output in the last decade. Recent bibliometric studies on individual mental health disorders have included ones on depression [3–5], suicide & self-harm [6, 7], Alzheimer’s disease [8–10] and addiction [11–14]. Most of these papers described the growth in the world literature, or in individual countries or regions. Many of them employed a similar methodology to the one used here, namely a comparison of research outputs with the disease burden, or with individual countries’ wealth.

### 1.3 The burden of mental health disorders

In this paper, we have defined mental health disorders to include all those listed by the World Health Organization (WHO) within the category II E "Mental and substance use disorders", with the addition of "Alzheimer’s and other dementias" (whose symptoms are primarily mental), and under category III B, "Self harm" which includes suicide. However, we excluded those whose cause is primarily genetic ("Autism and Asperger syndrome", "Childhood behavioural disorders", and "Idiopathic intellectual disability"). [This was the definition adopted for a recent project to map five European non-communicable disease research outputs in 2002–13 for the European Union [15, 16]. It was intended to exclude those mental health disorders that are not readily treatable as a result of research.]

In the OIC countries, the burden from mental health has been increasing steadily. This is shown by the percentage of the disease burden calculated by the WHO, and measured by Disability-Adjusted Life Years (DALYs). These are the sum of years lost because of early death, and years lived with a disability of varying severity, in a country. However, many of the data are unlikely to be reliable, particularly for 16 of the OIC African countries, whose data are marked by the WHO as being in the least good category. Table 2 shows data for 2000 and 2015 for 11 of the OIC countries, the group as a whole, and for three other sets of countries as comparators: Canada and the USA; the 27 Member States of the European Union plus Iceland, Norway, Switzerland and the UK (EUR31); and the Rest of the World (RoW).

**Table 2:** Percentages of total DALYs from mental disorders in some OIC countries in 2000 and 2015, the ratio between them, and corresponding percentages for other country groups.

*Country*	*ISO2*	*2000*	*2015*	*ratio*
Qatar	*QA*	12.0	17.5	*1*.*46*
United Arab Emirates	*AE*	14.0	16.6	*1*.*18*
Iran	*IR*	9.7	12.7	*1*.*31*
Turkey	*TR*	9.0	12.0	*1*.*34*
Tunisia	*TN*	9.9	11.8	*1*.*19*
Saudi Arabia	*SA*	8.8	11.6	*1*.*31*
Lebanon	*LB*	8.2	10.6	*1*.*28*
Malaysia	*MY*	9.2	10.4	*1*.*13*
Morocco	*MA*	8.3	10.1	*1*.*22*
Jordan	*JO*	7.8	10.0	*1*.*29*
Algeria	*DZ*	7.4	9.0	*1*.*21*
**OIC total**	***OIC***	**3.5**	**5.0**	***1*.*44***
Canada and USA	*CA*,*US*	13.8	17.8	*1*.*29*
Europe 31	*EUR*	11.3	13.7	*1*.*21*
Rest of the World	*RoW*	6.2	8.2	*1*.*32*

Data from the World Health Organization (WHO): http://www.who.int/healthinfo/global_burden_disease/estimates/en/index2.html

The mental health disorders burden is clearly increasing globally, but rather more rapidly in the OIC countries than in the other three geographical groups. Within the OIC, two countries (Qatar and the United Arab Emirates (UAE)) now have a greater relative mental health disorders burden than the European average, and all the ones listed have a higher percentage burden than the mean for the Rest of the World. The prevalence of any psychiatric disorder in Turkey has generally been found to be lower than in western countries (although somewhat higher than in Asian countries), as was shown in a collaborative international study [17, 18]. More recent epidemiological studies in the OIC countries revealed 12-month prevalence rates that were higher than those of Turkey (13.6% in Iraq and 20.2% in Saudi Arabia) [19, 20]. Although their incidence was relatively low then, it has since grown more rapidly than in most other OIC countries (Table 2).

## 2 Methodology

### 2.1 Formation of the database

Articles and reviews were identified in the Web of Science (WoS) by means of a complex filter based on 184 named mental disorder journals such as:

*Addiction* or *Behavior Genetics* or *Cognitive Therapy and Research* or *Depression and Anxiety* or *Eating Disorders* or *Forum der Psychoanalyse*

and 236 title words, or phrases, such as:

addict* or *bipolar* or *carbamazepine* or dement* or eating-disorder* or *frigidity* or *gambling* or *hysteria* or *inject-drugs*

which included the names of disorders, and drugs and other measures used to treat them. There was also a negative search statement designed to exclude papers in non-relevant subject areas, such as:

*AGRONOMY* or BUSINESS* or *CLASSICS* or *DEMOGRAPHY* or *ECOLOGY* or *FISHERIES* or GEO* or HISTORY*.

(This search strategy is reproduced in the S1 Appendix) Papers were selected if they had at least one address from an OIC country, see Table 1, and were published in the ten-year period, 2008–17. There was no language restriction. The filter was applied to the Science Citation Index-Expanded, the Social Sciences Citation Index, the Conference Proceedings Citation Index–Science and the Emerging Sciences Citation Index. The bibliographic details of the identified papers were downloaded to a series of text files, and then combined to form a single spreadsheet. [Individual inspection was used to remove non-relevant papers containing the words *drug* or *drugs*, and some containing the word *alcohol* which were concerned with the enhanced risks of cancer or with chemistry, respectively.] We calibrated the filter by marking samples of papers that it had identified as relevant, not relevant, or marginal [21], and determined the precision (specificity) as 0.74 and the recall (sensitivity) as 0.79. This meant that the true total of OIC papers was 0.74 / 0.79 = 0.94 times the apparent total.

We also compared the mental health disorders research outputs of the OIC countries with their overall biomedical research outputs in the same years. These were selected by means of a complex filter based on address terms [22], such as:

*AIDS* or *BETHESDA* or CHILD* or *DAIICHI* or EPIDEM* or *FAMILY*

so as to show the percentage of their biomedical research papers that were about mental health disorders. This comparison was based on integer counts of countries.

### 2.2 Geographical and subject analysis, and comparison with mental health disorders burden

The addresses on the papers were parsed by means of a Visual Basic Applications (VBA) program to show the fractional counts of the countries. [For example, a paper with two Turkish addresses and one from France would be classed as TR = 0.67, FR = 0.33.] We are aware that this allocation of credit to different countries may well mis-state the relative importance of the several contributions, but there is really no practical alternative. There are some papers with large numbers of authors (and addresses) and it would seem unreasonable to give each country (or author) full credit for the whole paper.

We also applied two special filter programs to the papers’ titles and journal names in order to classify them by the mental health disorder(s) investigated by the authors, and by the type of research (*e*.*g*., genetics, drugs). These are listed, with the codes used in this paper, in Table 3. These outputs were cross-tabulated by the fractional counts of the leading OIC countries so as to show which ones were relatively specialising in particular subject areas. We also compared the OIC outputs on individual mental health disorders with the relative DALY burden from them in 2010.

**Table 3:** List of mental health disorders and research types (or domains) subject to analysis, and their trigraph and tetragraph codes.

*Mental disorder*	*Code*	*Research Type*	*Code*
Addiction	ADD	Diagnosis	DIAG
Alcohol abuse	ALC	Drug treatments	DRUG
Alzheimer’s & other dementias	ALZ	Epidemiology	EPID
Anxiety and panic	ANX	Genetics	GENE
Bipolar disorder	BIP	Prognosis	PROG
Unipolar depression	DEP	Other (non-drug) treatments	TREA
Eating disorders	EAT		
Schizophrenia	SCH		
Suicide and self-harm	SUI		

A further analysis was of the amount of research in health systems. Compared with the research on individual disorders, this is not an easily described subject area. We adopted this definition [23]:

A mental health system is defined as the structure and all the activities whose primary purpose is to promote, maintain or restore mental health. It includes all organizations and resources that focus on improving mental health, and it is commonly used as synonymous with mental health services.

We created two lists of paper title words: one of the systems being examined (*e*.*g*., burden, program, service), and the other their geographical or operational context (*e*.*g*, Egyptian, rural). Papers were selected from the main database if they had a title word from each of these two lists, or were in the *International Journal of Mental Health Systems*.

In order to put the research outputs of the different countries in context, they were plotted against the Gross Domestic Product (GDP) of the leading OIC states as this normally gives a good correlation, at least for rich research-active countries [24]. Because the outputs of the leading OIC states had been growing quite rapidly and because in 2015 the WoS was expanded to process many additional journals published out with North America and Western Europe, we used outputs in 2015–17, and compared them with GDP values for 2014. However, because the OIC countries varied greatly in the percentage of all DALYs attributable to mental disorders (as we defined them), we multiplied their GDP values by this percentage.

### 2.3 International collaboration

We also wished to see the amount of international collaboration in mental health disorders research by the OIC countries, and which third countries they favoured as partners. For this purpose, we calculated the fractional count contributions of the leading OIC countries to their papers, and the corresponding contributions by other OIC countries, by Canada + the USA, by the EUR31 countries, and by the Rest of the World. For the individual non-OIC countries, we counted their integer tallies as a percentage of the total of the OIC papers with non-OIC contributions, and compared this with the percentage presence of each country in mental health disorders research, after excluding OIC papers, for each of two five-year periods, 2008–12 and 2013–17. [For example, in 2013–17, there were 780 British contributions to the total of 3326 papers that had contributions by non-OIC co-authors, or 23.45%, but the British presence in mental health disorders research in these years was 11.6%, so evidently the UK was preferred as a partner by a factor of 23.45/11.6 = 2.02. Many other countries would have been less preferred, with a factor of less than unity, for example China, with a factor of 6.73%/7.13% = 0.94 in these five years.]

### 2.4 Citation counts

There are two commonly-used measures of the influence of research papers. One is the importance of the journal in which they are published, as measured by the Clarivate Journal Impact Factor (JIF). We tabulated the mean JIF for the papers from 16 leading OIC countries, both for all their papers, and for their domestic ones (*i*.*e*., without international collaboration). [This gives a fairer estimate of the work of the local researchers in each country.] The second measure is the number of citations received from WoS journals, represented by the five-year citation scores (Actual Citation Impact, ACI) for the papers from the six years, 2008–13. These were determined from the WoS and copied and pasted to the file of papers. The citation counts were multiplied by the fractional contributions of each country to each paper, and then averaged. The mean value was also determined for domestic-only papers from each country.

The papers were also ranked by their ACI values, and the top 1% and 5% identified; they were cited respectively 59 and 26 times or more. (There were 386 papers with 26 citations or more, which is 386/7342 = 5.26%). The percentages of each country’s citable papers that received these numbers of citations or more were then calculated as percentages, divided by the average for the OIC of 1.01% or 5.26%, and then multiplied by 100 to give a "world scale" value [25]. This gives an alternative ranking of the OIC countries, although this is usually similar to that based on mean ACI values. We thus determined six separate indicators of the papers’ influence, and the countries were then ranked on each one, and ordered in the table by the mean value of these rankings.

## 3 Results

### 3.1 Outputs overall and by country

In the decade, 2008–17, there were a total of 17,920 papers in our database, and the total OIC fractional contribution was 15,170 papers. The difference of 2750 papers represented the contributions of other countries (15% of the total). The output grew at an annual rate of 14%, which is very high compared with that of the world in the same period (6.4%) or that of the USA (4.6%). There was a jump in output in 2015 when the Emerging Sciences Citation Index was established. It led to an increase in the numbers of OIC countries whose journals were covered in the WoS from ten to 18.

In the same ten-year period, the OIC countries published 478,038 biomedical research papers, which was 7% of the world total. Their mental health disorders output equalled 3.7% of their biomedical research output. However, the precision of the filter (p = 0.74) was lower than its recall (r = 0.79), so that the corrected total was only 3.5%. This compares with the disease burden of about 4.7% in those years.

Table 4 shows the fractional outputs of all the OIC countries, and the growth rate for the leading ten. [Six countries had no output during the decade.] Some countries, notably Malaysia, Jordan, Saudi Arabia and Iran, had outputs that grew at over 20% per year, but that of Kuwait actually *declined* over the decade (from 9.4 per year in 2008–10 to 5.7 per year in 2015–17).

**Table 4:** Outputs of mental health disorders research papers in the WoS, 2008–17, from the OIC countries, fractional counts, (for ISO codes, see Table 1).

*ISO2*	*N*	*AAPG*	*ISO2*	*N*	*AAPG*	*ISO2*	*N*	*ISO2*	*N*	*ISO2*	*N*
***OIC***	**15170**	**14.0**	*AE*	124	15.5	*KZ*	24.5	*AZ*	8.0	*GM*	2.2
*TR*	7073	9.1	*ID*	109	28.5	*CM*	23.8	*TG*	7.8	*NE*	2.1
*IR*	3621	20.3	*UG*	107	15.5	*DZ*	22.7	*BF*	7.7	*SL*	1.2
*MY*	813	25.3	*MA*	88.9	8.8	*SY*	16.4	*YE*	7.2	*ML*	1.1
*EG*	641	18.3	*KW*	68.0	-7.9	*BH*	15.6	*PS*	6.4	*GA*	1.0
*PK*	628	19.3	*QA*	61.2	29.6	*KG*	12.4	*SN*	6.4	*GY*	0.7
*NG*	477	9.6	*BD*	56.7	29.6	*CI*	10.9	*MZ*	6.3	*SR*	0.6
*SA*	469	21.2	*IQ*	49.1	15.2	*SD*	8.6	*UZ*	5.8	*TJ*	0.5
*TN*	210	9.5	*OM*	31.1	11.6	*LY*	8.4	*AF*	5.6	*TD*	0.4
*LB*	168	15.1				*BN*	8.2	*BJ*	2.9	*SO*	0.3
*JO*	137	24.5				*AL*	8.1	*GN*	2.4	*MR*	0.1

AAPG = Annual Average Percentage Growth. (Values > 2.0 x mean of 14.4%, tinted green; if > 1.414 x mean, tinted pale green; if < 0.707 x mean, tinted pale yellow; if < 0.5 x mean, tinted pink.)

**Fig 1:**
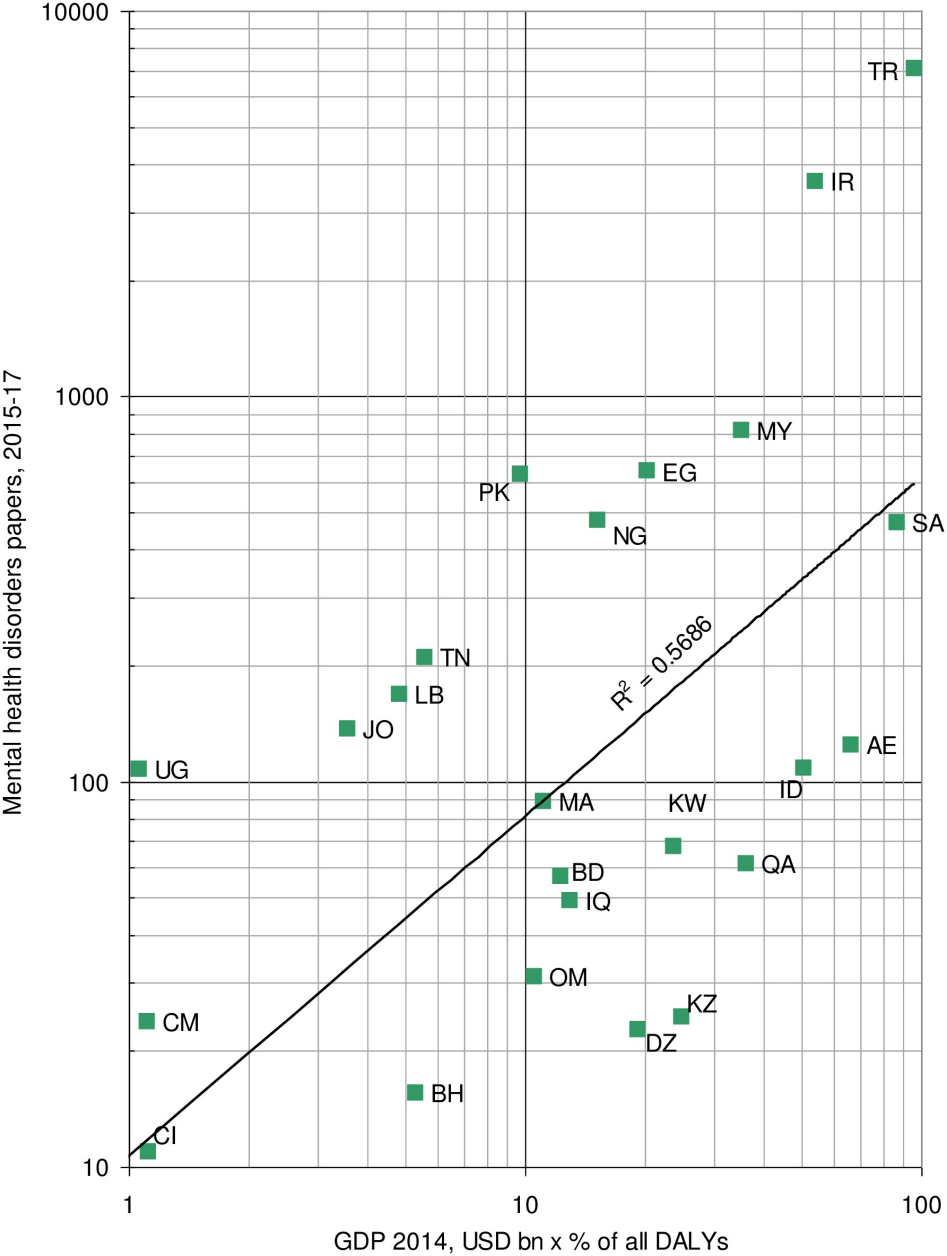
OIC country outputs of mental disorders papers, 2015–17 compared with their wealth multiplied by the percentage of all DALYs in 2015 from mental disorders.

The outputs of the 24 leading OIC countries in 2015–17 are plotted against their GDP in 2014, multiplied by the percentage of all DALYs in 2015 that are attributable to mental health disorders, in Fig 1. Although there is clearly a positive correlation, the spots for the countries are in two main groups, with eight countries publishing much more than expected, notably Pakistan (PK), Iran (IR) and Turkey (TR), and ten of the others, much less. Among these are some of the richest countries, with a high mental health disorders toll, such as Qatar (QA), United Arab Emirates (AE), Kuwait (KW) and Bahrain (BH), which are clearly not doing enough relevant research.

### 3.2 International collaboration

**Fig 2:**
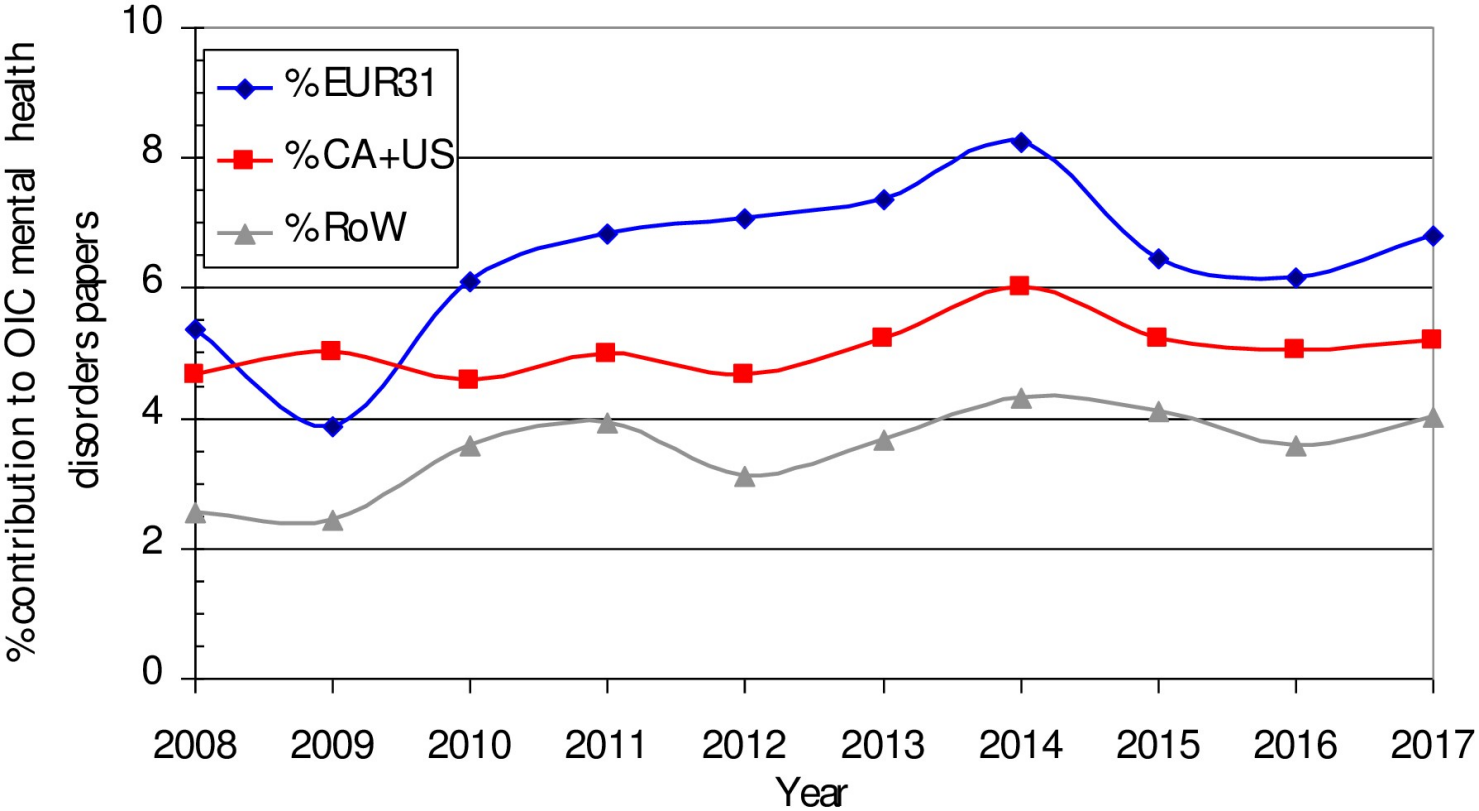
The contributions of non-OIC countries to WoS OIC mental disorders research papers, 2008–17.

**Fig 3:**
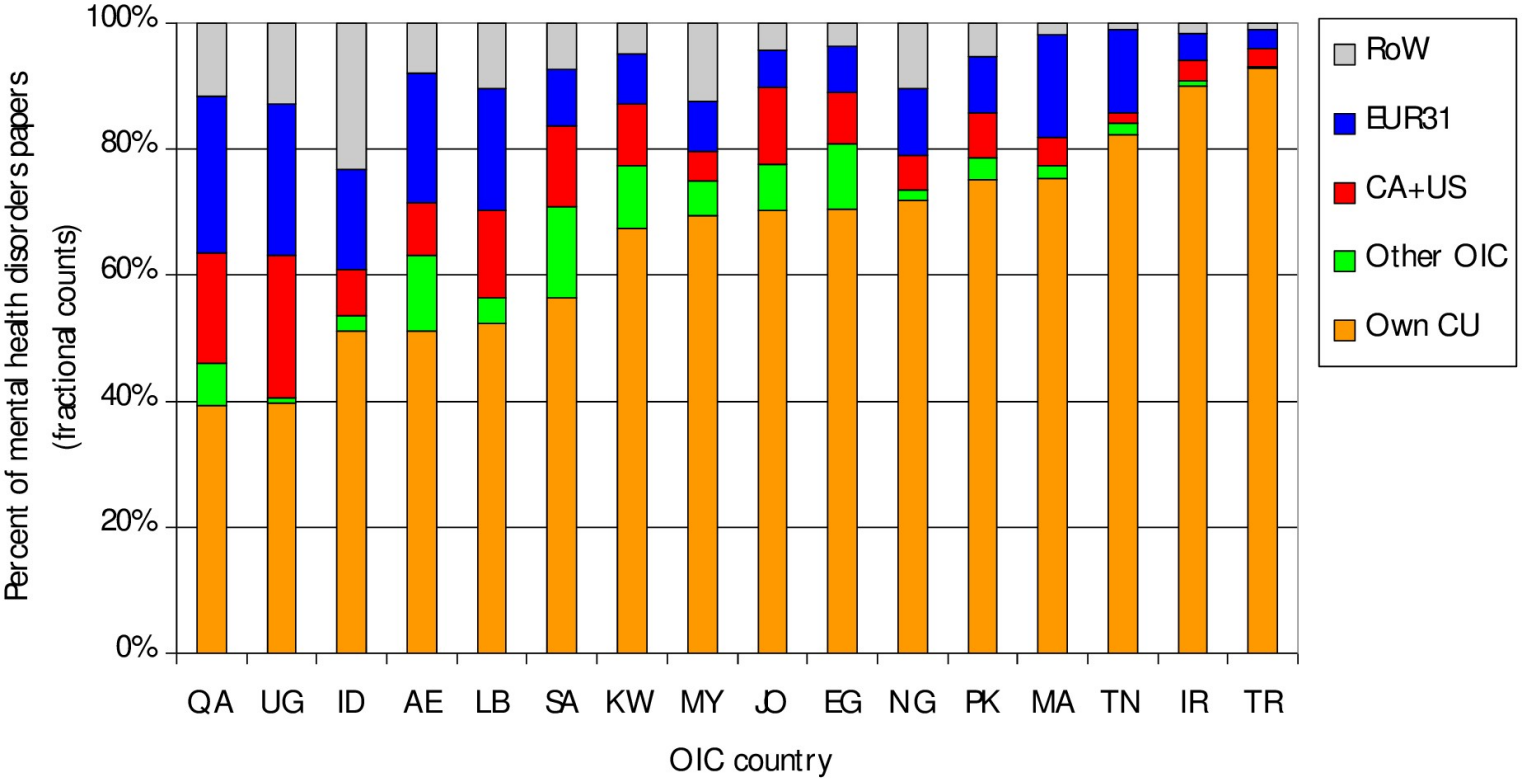
Collaboration in OIC mental disorders research with others in the OIC, Canada + USA, EUR31, and the Rest of the World (RoW).

Fig 2 shows the contributions of other countries, divided into three groups (Canada + the USA; the EUR31 countries; and the Rest of the World) to the OIC papers. The amount of collaboration with "third" countries has not increased much over time, especially with Canada and the USA. However, it is much higher for some of the OIC countries, see Fig 3, but much lower for Turkey (TR) and Iran (IR).

Table 5 shows the amount of collaboration between the OIC countries and other individual countries in two periods, and whether they are preferred, or non-preferred, as partners. Some of the cells are tinted to show where the ratios differ significantly from unity. For some of these countries these ratios have been increasing, but there are many exceptions, and China and South Korea have become less preferred as partners in the period 2013–17. Although most European countries are preferred as partners, the three most-preferred partner countries are all non-European, and are from different continents: South Africa (ZA), India (IN) and Mexico (MX).

**Table 5:** Collaboration in mental health disorders research between the OIC countries as a group with individual third countries in two quinquennia, 2008–12 and 2013–17 and percentages of total non-OIC papers (1360 and 3326, respectively; % OIC), compared with the countries’ percentage presence in world mental health disorders research in the same years (WoS, %), and ratio between them, all integer counts.

*Countries*	*ISO2*	*WoS*, *%*		*Within OIC set*		*OIC set*, *%*		*OIC/WoS*	
		*08–12*	*13–17*	*08–12*	*13–17*	*08–12*	*13–17*	*08–12*	*13–17*
USA	*US*	41.1	37.9	558	1350	41.0	40.6	*1*.*00*	*1*.*07*
UK	*UK*	10.9	11.6	332	780	24.4	23.5	2.23	2.02
Australia	*AU*	6.03	7.25	165	421	12.1	12.7	2.01	1.75
Germany	*DE*	8.25	7.61	136	320	10.0	9.62	1.21	1.27
Canada	*CA*	6.46	6.77	110	308	8.09	9.26	1.25	1.37
France	*FR*	3.71	3.55	134	223	9.85	6.70	2.65	1.89
Netherlands	*NL*	4.43	4.65	111	244	8.16	7.34	1.84	1.58
Italy	*IT*	4.58	4.83	84	237	6.18	7.13	1.35	1.48
Switzerland	*CH*	2.19	2.33	90	206	6.62	6.19	3.02	2.66
China	*CN*	3.68	7.13	74	224	5.44	6.73	1.48	*0*.*94*
India	*IN*	1.29	2.07	80	202	5.88	6.07	4.55	2.93
Spain	*ES*	3.89	4.11	76	211	5.59	6.34	1.44	1.54
Japan	*JP*	3.61	3.40	90	158	6.62	4.75	1.83	1.40
Belgium	*BE*	1.53	1.65	65	144	4.78	4.33	3.13	2.63
Brazil	*BR*	2.62	2.71	54	155	3.97	4.66	1.52	1.72
Sweden	*SE*	2.44	2.82	53	143	3.90	4.30	1.60	1.52
South Africa	*ZA*	0.58	0.76	45	135	3.31	4.06	5.73	5.34
Austria	*AT*	0.93	0.94	58	77	4.26	2.32	4.60	2.46
Israel	*IL*	1.48	1.42	51	73	3.75	2.19	2.53	1.54
Mexico	*MX*	0.52	0.57	44	79	3.24	2.38	6.20	4.19
South Korea	*KR*	1.82	2.45	38	74	2.79	2.22	1.53	*0*.*91*

(Cells where this ratio > 2.0 tinted green; > 1.41 tinted pale green. Values in *italic type* are **not** statistically significant at p < 0.05.)

### 3.3 Distribution of papers among mental health disorders and research domains

**Fig 4:**
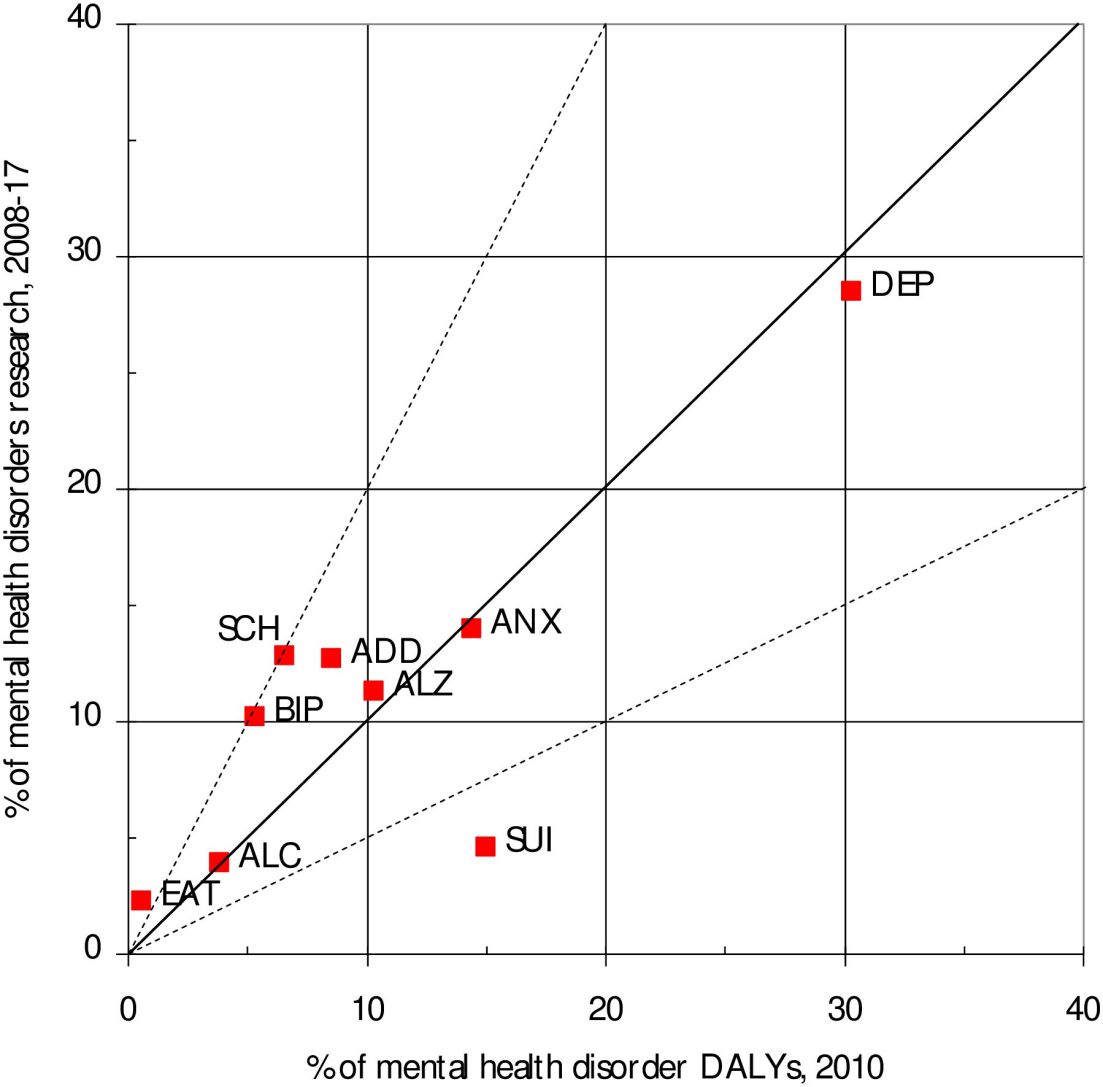
OIC mental disorders research outputs, 2008–17, compared with their mental disorders percentage burden in DALYs.

Fig 4 shows the distribution of papers among the leading mental health disorders in 2008–17 compared with the burden of disease from these different disorders in 2010. In this figure, the percentages of research in each of the nine disorders have been scaled up so that their sum equals 100% of the total. The diagonal line shows equivalence, and the dashed lines show where research outputs are twice, or half, the amount that would represent equivalence. Depression (DEP) is clearly the main disorder both in terms of research and disease burden, and the amount of research appears to be appropriate. However suicide and self harm (SUI) seems to be under-researched by a factor of three, whereas schizophrenia (SCH) and bipolar disorder (BIP) appear to attract about twice as much attention from OIC researchers as their disease burden would warrant.

The relative commitment of the leading 16 OIC countries to research on the different mental health disorders is shown in Table 6. Many of the cells are tinted to show values notably above (green and light green) or below (yellow and pink) unity. Depression (DEP), the main subject for research, is actively researched by Pakistan (PK) and Uganda (UG). Because alcohol is prohibited or discouraged in many Muslim countries, there is a big disparity in the amount of research that it attracts. It is very high in Uganda, the United Arab Emirates (AE) and Lebanon (LB), but very low in Kuwait (KW), Morocco (MA), Qatar (QA), Jordan (JO) and Tunisia (TN).

**Table 6:** The relative commitment to research on each of nine mental health disorders by the 16 leading OIC countries, fractional counts.

	*ADD*	*ALC*	*ALZ*	*ANX*	*BIP*	*DEP*	*EAT*	*SCH*	*SUI*
*TR*	**0.58**	*0*.*87*	**0.71**	**1.13**	**1.22**	*0*.*98*	1.26	**1.13**	*1*.*11*
*IR*	**1.66**	*0*.*97*	*1*.*00*	**1.27**	*0*.*95*	*1*.*03*	*0*.*89*	**0.74**	**0.74**
*MY*	**1.39**	*0*.*92*	**1.39**	*0*.*86*	**0.54**	*0*.*93*	*1*.*31*	*0*.*98*	*1*.*23*
*EG*	*0*.*93*	*0*.*59*	**2.00**	**0.38**	**1.50**	*0*.*98*	*0*.*67*	*1*.*16*	0.46
*PK*	0.64	*0*.*72*	*0*.*73*	1.30	*1*.*11*	**1.64**	*1*.*05*	*0*.*96*	*1*.*18*
*NG*	0.68	*1*.*21*	0.65	*0*.*87*	**0.31**	*1*.*04*	*0*.*50*	*1*.*23*	*1*.*00*
*SA*	0.67	*0*.*72*	**1.57**	*0*.*77*	*0*.*85*	*1*.*18*	0.12	*0*.*78*	0.46
*TN*	**0.27**	*0*.*48*	1.49	**0.23**	**2.21**	**0.45**	*0*.*31*	**2.69**	*1*.*23*
*LB*	**1.83**	2.10	*0*.*49*	*1*.*15*	*0*.*70*	*0*.*97*	*2*.*05*	*0*.*61*	*0*.*64*
*JO*	*1*.*35*	*0*.*32*	*0*.*71*	*1*.*27*	**0.09**	*1*.*24*	*0*.*53*	*0*.*86*	*0*.*43*
*AE*	*0*.*96*	**3.68**	*0*.*81*	*1*.*29*	*0*.*66*	*1*.*05*	2.60	*0*.*56*	*0*.*71*
*ID*	*1*.*47*	*1*.*19*	*1*.*16*	*0*.*50*	*0*.*51*	0.53	*0*.*91*	*0*.*57*	*1*.*05*
*UG*	0.22	**4.12**	0.23	0.29	*0*.*37*	1.42	*0*.*00*	0.17	**2.56**
*MA*	*1*.*65*	*0*.*05*	*0*.*81*	*0*.*87*	*0*.*88*	*0*.*64*	*0*.*98*	*1*.*00*	*0*.*94*
*KW*	*0*.*53*	*0*.*00*	*0*.*36*	*1*.*33*	*0*.*74*	*0*.*78*	*2*.*57*	*1*.*41*	*0*.*59*
*QA*	*0*.*18*	0.17	*0*.*84*	*0*.*80*	*0*.*65*	*0*.*70*	*0*.*11*	*1*.*76*	0.43

(Codes in Tables 1 and 3. Cells with values > 2.0 tinted green; where > 1.414 tinted light green; where < 0.717 tinted pale yellow; where < 0.5 tinted pink.) Values not statistically significantly different (with p < 0.05) from unity in *italics*, values with p < 0.005 in **bold**).

Table 7 shows the relative commitments of the leading 16 countries to research in the different domains listed in Table 3. Drug treatment (DRUG) is by far the most popular type of research and is actively supported in Egypt (EG) and Iran (IR). The latter, and Turkey (TR), are also active in non-pharmaceutical treatments (TREA), whereas research on these is almost ignored in most of the other OIC countries, and the combined OIC output is only 3.7% of the total compared with 5.8% in Europe [16]. Epidemiology (EPID) is researched fairly equally in all the countries listed here, but five of them do noticeably more than expected (Indonesia, ID; Lebanon, LB; Uganda, UG; Tunisia, TN and Malaysia, MY). Genetics only represents 6.3% of the total, much lower than in Europe (16%) [16]. Papers on health services research (n = 526) represented 2.8% of the total. This is much higher than their presence in world mental health disorders papers (0.95%). The countries most involved were Iran (81), Nigeria (69.7), and Turkey (42.8 papers).

**Table 7:** Relative commitment within mental health disorders research to work on six research types (codes in Table 3) by the leading 16 countries (codes in Table 1), 2008–17, fractional counts.

*ISO2*	*DIAG*	*DRUG*	*EPID*	*GENE*	*PROG*	*TREA*	*Frac ct*
*TR*	*0*.*99*	0.92	0.76	*0*.*93*	0.78	*1*.*03*	7073
*IR*	*1*.*00*	1.44	0.84	*1*.*00*	*1*.*00*	1.42	3621
*MY*	*0*.*96*	0.85	1.46	*1*.*31*	1.45	*0*.*89*	813
*EG*	*0*.*70*	1.81	0.75	*0*.*90*	*0*.*82*	*0*.*63*	641
*PK*	*0*.*79*	*1*.*11*	*1*.*09*	0.59	*1*.*04*	*0*.*72*	628
*NG*	*0*.*50*	0.55	*1*.*09*	0.42	*1*.*14*	*0*.*70*	477
*SA*	*1*.*45*	1.38	*1*.*08*	*1*.*18*	*1*.*01*	0.43	469
*TN*	*0*.*74*	0.61	1.50	2.42	*0*.*81*	0.21	210
*LB*	*0*.*39*	0.55	1.57	*0*.*77*	*1*.*20*	*0*.*77*	168
*JO*	*0*.*31*	*0*.*72*	*1*.*31*	*0*.*95*	*1*.*34*	*0*.*19*	137
*AE*	*1*.*14*	*0*.*59*	*1*.*28*	*0*.*63*	*1*.*26*	*0*.*93*	124
*ID*	*0*.*23*	0.38	1.75	*1*.*47*	*0*.*63*	*0*.*61*	109
*UG*	*0*.*94*	0.14	*1*.*51*	*0*.*54*	*1*.*56*	*1*.*22*	107
*MA*	*0*.*93*	*0*.*68*	*1*.*03*	*1*.*34*	*0*.*54*	*0*.*04*	88.9
*KW*	*1*.*43*	*0*.*70*	*0*.*83*	*1*.*51*	*0*.*86*	*0*.*00*	68.0
*QA*	*2*.*15*	*0*.*74*	*0*.*82*	*0*.*96*	*1*.*12*	*0*.*44*	61.2
*Total*	460	3094	1766	1125	753	670	17920

(Cells where this ratio > 2.0 tinted green; > 1.41 tinted pale green; < 0.71 tinted pale yellow; < 0.50 tinted pink. Values not statistically significantly different (with p < 0.05) from unity in *italics*.)

### 3.4 The citation performance of the OIC countries

The last analysis is of the journal impact factors of all the papers, and of the citations to their 2008–13 papers, for the 16 leading OIC countries. The results are presented in Table 8, with the countries ranked by the mean of the six rankings. The best-performing country is Lebanon, followed by Uganda. Both countries collaborate extensively internationally (see Fig 3), and this has contributed to their higher scores on JIF and ACI for all their papers than for their domestic ones. However, in terms of domestic-only papers, Egypt is ranked highest on both indicators. It is notable that the performance of Iran (IR) is substantially better than that of Turkey, on all six indicators, although both countries collaborate internationally very little, see Fig 3.

**Table 8:** Journal Impact Factors for all and domestic mental health disorders research papers from 16 leading OIC countries; also citation performance (five-year counts, fractional country counts) (ACI all and ACI dom), and presence in the top 1% and 5% (59, 26 cites or more) showing World-Scale (W.S.) values (relative to norm of 100).

*Country*	*JIF all*	*JIF dom*	*Citable*	*Cites*	*ACI all*	*ACI dom*	*W*.*S*. *1%*	*W*.*S*. *5%*
*LB*	3.61	1.95	74.4	768	10.32	7.83	*113*	*150*
*UG*	3.45	1.35	44.1	543	12.33	7.46	*97*	229
*AE*	2.52	2.08	55.8	473	8.49	4.80	*44*	*115*
*EG*	2.45	2.13	258	2099	8.15	8.17	*13*	70
*QA*	3.22	1.97	17.0	141	8.29	3.00	*194*	59
*IR*	1.96	1.77	1226	9854	8.04	7.59	32	*108*
*SA*	2.28	1.34	150	1127	7.52	6.00	*142*	*106*
*MY*	2.15	1.71	287	1999	6.95	6.12	*86*	77
*JO*	2.22	1.48	38.1	340	8.90	7.79	*19*	58
*ID*	3.19	2.00	27.1	225	8.28	3.80	*0*	17
*TN*	1.82	1.34	100	656	6.54	5.27	*49*	*88*
*NG*	2.82	1.62	233	1233	5.29	4.26	*34*	25
*PK*	1.86	1.20	198	1262	6.39	5.43	*30*	71
*TR*	1.51	1.27	3337	17200	5.15	4.84	16	39
*KW*	1.72	1.49	47.5	227	4.77	5.11	*0*	0
*MA*	2.17	0.88	38.9	130	3.34	2.13	*0*	4

(Cells where this ratio > 2.0 tinted green; > 1.41 tinted pale green; < 0.71 tinted pale yellow; < 0.50 tinted pink. Values of W.S. that are not statistically significant (with p < 0.05) in *italics*.)

## 4 Discussion

### 4.1 Outputs of the countries

The 57 OIC countries are increasing their outputs of mental health disorders research papers quite rapidly. From 2008 to 2017, their output grew at 14.4% per annum compared with 6.4% for the world total. This shows their appreciation of the growing burden of mental health disorders for their respective populations, which accounted for 5% of their total health burden in 2015, having increased from just 3.5% in 2000. However, many of the OIC countries in the Middle East region suffered much more from mental health disorders, particularly Qatar (QA) and the United Arab Emirates (AE) whose burden in DALYs was greater than 16% in 2015. The oil-rich countries–Saudi Arabia (SA), UAE, Qatar, Kuwait (KW), Iraq (IQ), and Nigeria (NG)–are the OIC countries with the highest relative burden from mental health disorders (Table 2), so really need to do more to build their research capacity and capability in mental health.

### 4.2 International collaboration

There is also a wide variation in the tendency of the OIC countries to collaborate internationally (Fig 3). This is a prime pre-condition for a country to be able to take part in high-level, internationally-impactful research. Some, such as Uganda (UG), the UAE and Lebanon (LB) collaborate actively: this has also led to a better citation record (Table 8). Iran has found itself rather scientifically isolated because of the international sanctions in place [26, 27], but Turkey’s isolation from other countries in this research area, as well as in cancer [28] is less understandable. It may be due to the way psychiatric research is evaluated in the country, with counts of WoS papers being used to gain academic posts, without consideration of their clinical impact. Its poor citation record as a result suggests that new policies for international engagement of this now strongly research-active country may be necessary. The OIC group as a whole appear to favour partnerships with both European and other non-EU countries, except those in East Asia. [The generally high values of preference are largely because integer counting has been used, so that papers with several non-OIC countries among the addresses count for all of them.]

### 4.3 Comparison of research with the disease burden

The output of OIC mental health disorders papers, as a percentage of their biomedical research output (3.5%), was somewhat lower than their disease burden at about 5.0% of all DALYs. However, it was much closer than the corresponding results for either Canada and the USA (6.9% *cf*. 16.5%) or for the EUR31 countries (6.2% *cf*. 12.9%). This shows that the OIC countries are taking mental health seriously in terms of research, at least in comparison with North America and Europe, where it appears to be neglected [16].

The distribution of research by disorder correlates fairly well with the overall disease burden from individual disorders (Fig 4); r^2^ = 0.69. Depression is the main mental disorder and attracts the most research. The main exception is research into suicide and self-harm (SUI). The figure indicates that these countries are doing only about one third as much research as the burden would appear to justify. This may in part be because of social attitudes, so that it is stigmatised more than depression, and the fact that suicide is regarded as a sin by Islam. It may also be occasioned by the thought that not much can be done about it, but recent results show that well-designed programmes can make a difference to outcomes [29–31]. The success of charitable organisations such as the Samaritans in the UK and Ireland also shows that practical help can be given by trained volunteers who listen on the telephone to people with suicidal thoughts.

It is also of note that alcohol abuse is apparently receiving an appropriate amount of attention at 3.9% of the total on an adjusted basis. This compares favourably with the situation in Europe, where research on alcohol misuse is grossly neglected [11]. It is relatively most strongly researched in Uganda (UG), the UAE (AE) and Lebanon (LB). The legal situation for the sale of alcohol in the OIC group varies, and it is prohibited to all persons in 11 of them, and to Muslims in four more. Average *per caput* annual consumption is only 3.0 litres of ethanol in the OIC states compared with 7.6 in others (WHO, 2010). Alcohol is drunk widely in Uganda (9.8 l/yr) and Lebanon (6.7 l/yr), and available with a licence in the UAE (4.3 l/yr). However, the disease burden in 2010 was quite small in Uganda (0.2%) and in Lebanon (0.13%) suggesting that this particular area of mental health research is being appropriately addressed by individual countries within the OIC group.

Eating disorders (which in Europe mainly involve anorexia and bulimia) have a rather low burden in OIC group countries, with only 0.03% of the total DALYs. This is even lower in the 22 sub-Saharan African countries (average 0.013%) where high body mass index is often seen as a sign of good health; it is relatively higher in the others (average 0.092%). However, it does appear that anorexia is by no means absent from girls in Nigeria [32] and from young people in Turkey [33].

Drug treatment of mental health is the one most researched in the OIC group countries (Table 7). There appears to be rather little interest in non-pharmaceutical treatments except in Turkey (n = 273 papers) and Iran (n = 193). The main treatments researched were Cognitive Behavioural Therapy (CBT, 152 papers) and Electroconvulsive Therapy (ECT, 134 papers). Other treatments were surgery (for sleep apnoea, bariatric weight loss and cosmetic reasons; 47 papers), counselling (46), transcranial stimulation (40), and music (16) and play (12) therapy. There were also nine papers on couple/family/sex therapy.

### 4.4 Citation performance of countries

Papers from the OIC group were not well cited compared with those from their main partners. The overall average value of ACI was 8.0 cites in five years, but for the Netherlands, in comparison, it was 21.8; for the UK, 19.6 and for India, 19.4 cites. Lebanon (LB) and Uganda (UG) achieved the best performance among the OIC group (Table 8). This is likely to be because both countries collaborated extensively with Canada and the USA, and with the EUR31 countries (Fig 3), and were dominated by a few individuals. For example, of Uganda’s 92 citable papers (from 2008–13), 79 involved a foreign partner, of whom over half were from either the USA, the UK or the Netherlands. The leading Ugandan researchers were Professor Eugene Kinyanda of the MRC AIDS research unit in Entebbe and Professor Seggane Musisi of Makerere University in Kampala; together they contributed to 39 of these 92 papers. However, there were a total of 114 different Ugandan authors on these papers. Of Lebanon’s 148 citable papers, 100 were international, and these had a mean ACI of 33.6, compared with just 7.8 cites for the domestic papers. Lebanon’s citable output was dominated by the American University in Beirut, with contributions to 57 of the papers (39%). Again, there were a large number (190) of different Lebanese contributors to these papers.

Turkey’s citation performance is likely to be poor because 26% of its citable papers were in Turkish; they averaged only 1.7 cites per paper compared with 7.9 for the ones in English. So, the policy of the WoS in processing these non-English language journals has downgraded Turkey’s citation score. However, the Turkish-language journal, *Türk Psikiyatri Dergisi (Turkish Journal of Psychiatry)* also has English-language texts in the online version, and so is much better cited (ACI = 3.5). By contrast, Iran published almost exclusively in English and so its citation performance (mean ACI = 9.0) was superior. However, if research is to be used nationally to benefit patients, publications in the national language are likely to have more immediate influence than ones in English.

One potential cause of the papers from Turkey (and Iran) not being more influential is the lack of contestable funding in these countries. For example, in Turkey, of the 6667 domestic papers, only 563 (8.4%) acknowledged any financial support, and most of this (406 papers, 72%) was from university own funds. Only 77 papers (1.2%) acknowledged the support of TUBITAK, the Scientific and Technological Research Council of Turkey. In Iran, of the 3165 domestic papers, 1464 (46%) did acknowledge funding, but again, most of this (1314 papers, 90%) was from universities, and only 49 (1.5%) acknowledged the INSF, the Iran National Science Foundation. So it appears that in these two countries, there was very little contestable funding for mental health disorders research. Table 9 shows that the presence of either TUBITAK or INSF increased both JIF and ACI, but the effects were not dramatic. Papers without any funding acknowledgements performed less well than ones with one or more.

**Table 9:** Mean Journal Impact Factors (JIF) and Actual Citation Impact (ACI, five years) for Turkish and Iranian domestic mental health disorders papers with different sets of funding acknowledgements.

*Country*	*Funding*	*Papers*	*Mean JIF*	*Mean ACI*
Turkey	TUBITAK	77	2.21	8.8
Turkey	Other	486	2.03	8.6
Turkey	None	6104	1.18	4.5
Iran	INSF	49	2.90	9.4
Iran	Other	1495	1.86	8.6
Iran	None	1701	1.62	6.8

TUBITAK = Scientific and Technological Research Council of Turkey; INSF = Iran National Science Foundation.

### 4.5 Limitations of this study

The main limitation is that the filter for mental health disorders was not as good as we would have wished. For other medical subject areas we have usually been able to develop a filter with both precision and recall above 0.9. This was not possible for mental health disorders because many of them have "ordinary" words to describe them, such as "depression", which have several other meanings. We tried to avoid these by the removal of papers in a long list of non-medical subject areas but this was inevitably not complete.

A second limitation is that we have used only one bibliometric source, the WoS, in order to compile our own database. There will be additional relevant papers recorded in others, such as Scopus and MedLine. However, the different formats would have made analysis difficult, particularly for the comparison of mental health disorders research with all biomedical research, and the analysis of citations. The WoS is carefully curated, and in 2015 its journal coverage was extended to include many more journals published in "non-traditional" countries. Thus from 2008–14, it included papers in 100 journals published in OIC countries (primarily in Turkey, n = 37), but in 2015–17 it covered 235 such journals, with 98 from Turkey and 54 from Iran. However, the only languages spoken in OIC countries whose papers were covered were Turkish (n = 1598) and Malay (n = 11).

A third limitation is that we have not been able to measure the "quality" of the research papers other than through journal impact factors and counts of citations in the serial literature. The term "quality" is an elusive concept, and should include additional measures such as the citation of papers on clinical practice guidelines (CPGs), their citation on patents, and their citation on policy documents. However a particular difficulty with the research outputs from OIC countries is that few of them publish CPGs, and they do not take out many patents, so that these measures can really only be applied to documents from non-OIC countries, and very few OIC papers will be cited in this way.

### 4.6 Conclusions

Although the OIC countries as a group are publishing more research on mental health disorders, relative to their disease burden, than either Europe, or Canada and the USA, some individual countries are under-performing in view of their wealth and the rising disease burden that they face. More international collaboration would help, especially between different OIC countries which may share common social norms. In particular, suicide and self-harm are seriously under-researched, and lessons could be learned from countries where research-based methods of prevention have achieved some success.

## Supporting information

S1 Appendix The mental health disorders filter for use on the Web of Science. (DOCX)
