# Supplementary material for: Mental health disorders research in the countries of the Organisation of Islamic Cooperation (OIC), 2008–17, and the disease burden: Bibliometric study
Source: PLoS One. 2021 Apr 23;16(4):e0250414. doi: 10.1371/journal.pone.0250414 (PMC8064544; doi:10.1371/journal.pone.0250414)
Supplement: S1 Appendix — (DOCX) [file pone.0250414.s001.docx]

**S1 APPENDIX**

**THE MENTAL HEALTH DISORDERS FILTER FOR USE ON THE WEB OF SCIENCE**

SO=(ACTA-NEUROPSYCHIATRICA OR ACTA-PSYCHIATRICA-SCANDINAVICA OR ACTAS-ESPANOLAS-DE-PSIQUIATRIA OR ADDICTION-REVIEWS OR ADDICTION-SCIENCE-CLINICAL-PRACTICE OR ADDICTIVE-DISORDERS-THEIR-TREATMENT OR ADHD-ATTENTION-DEFICIT-AND-HYPERACTIVITY-DISORDERS OR ADICCIONES OR ADVANCES-IN-MENTAL-HEALTH OR AFRICAN-JOURNAL-OF-PSYCHIATRY OR ALCOHOLISM-TREATMENT-QUARTERLY OR ALZHEIMER-DISEASE-ASSOCIATED-DISORDERS OR ALZHEIMERS-DEMENTIA OR ALZHEIMERS-RESEARCH-THERAPY OR AMERICAN-JOURNAL-OF-ALZHEIMERS-DISEASE-AND-OTHER-DEMENTIAS OR AMERICAN-JOURNAL-OF-DRUG-AND-ALCOHOL-ABUSE OR AMERICAN-JOURNAL-OF-GERIATRIC-PSYCHIATRY OR AMERICAN-JOURNAL-OF-PSYCHIATRY OR AMERICAN-JOURNAL-ON-ADDICTIONS OR ANADOLU-PSIKIYATRI-DERGISI-ANATOLIAN-JOURNAL-OF-PSYCHIATRY OR ANIMAL-MODELS-FOR-MEDICATIONS-SCREENING-TO-TREAT-ADDICTION OR ANNALS-OF-CLINICAL-PSYCHIATRY OR ANNALS-OF-GENERAL-PSYCHIATRY OR ARCHIVES-OF-GENERAL-PSYCHIATRY OR ARCHIVES-OF-PSYCHIATRIC-NURSING OR ARCHIVES-OF-SUICIDE-RESEARCH OR ARCHIVES-OF-WOMENS-MENTAL-HEALTH OR ASEAN-JOURNAL-OF-PSYCHIATRY OR ASIA-PACIFIC-PSYCHIATRY OR ASIAN-JOURNAL-OF-PSYCHIATRY OR AUSTRALASIAN-PSYCHIATRY OR AUSTRALIAN-AND-NEW-ZEALAND-JOURNAL-OF-PSYCHIATRY OR BEHAVIOUR-RESEARCH-AND-THERAPY OR BIOMARKERS-OF-NEUROLOGICAL-AND-PSYCHIATRIC-DISEASE OR BIPOLAR-DISORDERS OR BMC-PSYCHIATRY OR BRAIN-RESEARCH-IN-ADDICTION OR BRITISH-JOURNAL-OF-PSYCHIATRY OR CANADIAN-JOURNAL-OF-PSYCHIATRY-REVUE-CANADIENNE-DE-PSYCHIATRIE OR CHILD-AND-ADOLESCENT-PSYCHIATRY-AND-MENTAL-HEALTH OR CHILD-PSYCHIATRY-HUMAN-DEVELOPMENT OR COGNITIVE-BEHAVIOUR-THERAPY OR COGNITIVE-NEUROPSYCHIATRY OR COGNITIVE-THERAPY-AND-RESEARCH OR COMPREHENSIVE-PSYCHIATRY OR CRISIS-THE-JOURNAL-OF-CRISIS-INTERVENTION-AND-SUICIDE-PREVENTION OR CULTURAL-PSYCHIATRY OR CURRENT-ALZHEIMER-RESEARCH OR CURRENT-OPINION-IN-PSYCHIATRY OR CURRENT-PSYCHIATRY-REPORTS OR CURRENT-PSYCHIATRY-REVIEWS OR DEMENTIA-AND-GERIATRIC-COGNITIVE-DISORDERS OR DEMENTIA-AND-GERIATRIC-COGNITIVE-DISORDERS-EXTRA OR DEMENTIA-INTERNATIONAL-JOURNAL-OF-SOCIAL-RESEARCH-AND-PRACTICE OR DEPRESSION-AND-ANXIETY OR DUSUNEN-ADAM-JOURNAL-OF-PSYCHIATRY-AND-NEUROLOGICAL-SCIENCES OR EARLY-INTERVENTION-IN-PSYCHIATRY OR EATING-DISORDERS OR ENCEPHALE-REVUE-DE-PSYCHIATRIE-CLINIQUE-BIOLOGIQUE-ET-THERAPEUTIQUE OR EPIDEMIOLOGIA-E-PSICHIATRIA-SOCIALE-AN-INTERNATIONAL-JOURNAL-FOR-EPIDEMIOLOGY-AND-PSYCHIATRIC-SCIENCES OR EPIDEMIOLOGY-AND-PSYCHIATRIC-SCIENCES OR EUROPEAN-ADDICTION-RESEARCH OR EUROPEAN-ARCHIVES-OF-PSYCHIATRY-AND-CLINICAL-NEUROSCIENCE OR EUROPEAN-CHILD-ADOLESCENT-PSYCHIATRY OR EUROPEAN-EATING-DISORDERS-REVIEW OR EUROPEAN-JOURNAL-OF-PSYCHIATRY OR EUROPEAN-NEUROPSYCHOPHARMACOLOGY OR EUROPEAN-PSYCHIATRY OR EVIDENCE-BASED-MENTAL-HEALTH OR FRONTIERS-IN-PSYCHIATRY OR GENERAL-HOSPITAL-PSYCHIATRY OR GLOBAL-MENTAL-HEALTH OR HARVARD-REVIEW-OF-PSYCHIATRY OR HEROIN-ADDICTION-AND-RELATED-CLINICAL-PROBLEMS OR HUMAN-PSYCHOPHARMACOLOGY-CLINICAL-AND-EXPERIMENTAL OR INDIAN-JOURNAL-OF-PSYCHIATRY OR INTERNATIONAL-CLINICAL-PSYCHOPHARMACOLOGY OR INTERNATIONAL-GAMBLING-STUDIES OR INTERNATIONAL-JOURNAL-OF-BIPOLAR-DISORDERS OR INTERNATIONAL-JOURNAL-OF-COGNITIVE-THERAPY OR INTERNATIONAL-JOURNAL-OF-EATING-DISORDERS OR INTERNATIONAL-JOURNAL-OF-GERIATRIC-PSYCHIATRY OR INTERNATIONAL-JOURNAL-OF-MENTAL-HEALTH-AND-ADDICTION OR INTERNATIONAL-JOURNAL-OF-MENTAL-HEALTH-NURSING OR INTERNATIONAL-JOURNAL-OF-MENTAL-HEALTH-SYSTEMS OR INTERNATIONAL-JOURNAL-OF-METHODS-IN-PSYCHIATRIC-RESEARCH OR INTERNATIONAL-JOURNAL-OF-PSYCHIATRY-IN-CLINICAL-PRACTICE OR INTERNATIONAL-JOURNAL-OF-PSYCHIATRY-IN-MEDICINE OR INTERNATIONAL-JOURNAL-OF-SOCIAL-PSYCHIATRY OR INTERNATIONAL-PSYCHOGERIATRICS OR ISRAEL-JOURNAL-OF-PSYCHIATRY-AND-RELATED-SCIENCES OR JAMA-PSYCHIATRY OR JMIR-MENTAL-HEALTH OR JOURNAL-OF-ADDICTION-MEDICINE OR JOURNAL-OF-ADDICTIVE-DISEASES OR JOURNAL-OF-AFFECTIVE-DISORDERS OR JOURNAL-OF-ALZHEIMERS-DISEASE OR JOURNAL-OF-ANXIETY-DISORDERS OR JOURNAL-OF-CHILD-ADOLESCENT-SUBSTANCE-ABUSE OR JOURNAL-OF-CHILD-AND-ADOLESCENT-PSYCHIATRIC-NURSING OR JOURNAL-OF-CHILD-AND-ADOLESCENT-PSYCHOPHARMACOLOGY OR JOURNAL-OF-CLINICAL-PSYCHIATRY OR JOURNAL-OF-CLINICAL-PSYCHOPHARMACOLOGY OR JOURNAL-OF-EATING-DISORDERS OR JOURNAL-OF-ECT OR JOURNAL-OF-GAMBLING-STUDIES OR JOURNAL-OF-GERIATRIC-PSYCHIATRY-AND-NEUROLOGY OR JOURNAL-OF-MENTAL-HEALTH OR JOURNAL-OF-MENTAL-HEALTH-POLICY-AND-ECONOMICS OR JOURNAL-OF-MENTAL-HEALTH-TRAINING-EDUCATION-AND-PRACTICE OR JOURNAL-OF-MOOD-DISORDERS OR JOURNAL-OF-NERVOUS-AND-MENTAL-DISEASE OR JOURNAL-OF-NEUROPSYCHIATRY-AND-CLINICAL-NEUROSCIENCES OR JOURNAL-OF-OBSESSIVE-COMPULSIVE-AND-RELATED-DISORDERS OR JOURNAL-OF-PERSONALITY-DISORDERS OR JOURNAL-OF-PSYCHIATRIC-AND-MENTAL-HEALTH-NURSING OR JOURNAL-OF-PSYCHIATRIC-PRACTICE OR JOURNAL-OF-PSYCHIATRIC-RESEARCH OR JOURNAL-OF-PSYCHIATRY-NEUROSCIENCE OR JOURNAL-OF-SOCIAL-WORK-PRACTICE-IN-THE-ADDICTIONS OR JOURNAL-OF-SUBSTANCE-ABUSE-TREATMENT OR JOURNAL-OF-THE-AMERICAN-ACADEMY-OF-CHILD-AND-ADOLESCENT-PSYCHIATRY OR JOURNAL-OF-THE-CANADIAN-ACADEMY-OF-CHILD-AND-ADOLESCENT-PSYCHIATRY OR JOURNAL-OF-TRAUMATIC-STRESS OR KLINIK-PSIKOFARMAKOLOJI-BULTENI-BULLETIN-OF-CLINICAL-PSYCHOPHARMACOLOGY OR LANCET-PSYCHIATRY OR NEUROBIOLOGY-OF-DEMENTIA OR NEUROPSYCHIATRIC-DISEASE-AND-TREATMENT OR NEUROPSYCHIATRIE OR NEUROPSYCHIATRY OR NEW-DIRECTIONS-FOR-CHILD-AND-ADOLESCENT-DEVELOPMENT OR NORDIC-JOURNAL-OF-PSYCHIATRY OR NPJ-SCHIZOPHRENIA OR NUTRITION-IN-PREVENTION-AND-MANAGEMENT-OF-DEMENTIA OR PERSONALITY-AND-MENTAL-HEALTH OR PERSONALITY-DISORDERS-THEORY-RESEARCH-AND-TREATMENT OR PERSPECTIVES-IN-PSYCHIATRIC-CARE OR PHARMACOPSYCHIATRY OR PRIMARY-CARE-COMMUNITY-PSYCHIATRY OR PROGRESS-IN-BRAIN-RESEARCH OR PROGRESS-IN-NEURO-PSYCHOPHARMACOLOGY-BIOLOGICAL-PSYCHIATRY OR PSYCHIATRIA-DANUBINA OR PSYCHIATRIA-POLSKA OR PSYCHIATRIC-CLINICS-OF-NORTH-AMERICA OR PSYCHIATRIC-GENETICS OR PSYCHIATRIC-QUARTERLY OR PSYCHIATRISCHE-PRAXIS OR PSYCHIATRY-AND-CLINICAL-NEUROSCIENCES OR PSYCHIATRY-AND-CLINICAL-PSYCHOPHARMACOLOGY OR PSYCHIATRY-INTERPERSONAL-AND-BIOLOGICAL-PROCESSES OR PSYCHIATRY-INVESTIGATION OR PSYCHIATRY-RESEARCH OR PSYCHIATRY-RESEARCH-NEUROIMAGING OR PSYCHOLOGICAL-MEDICINE OR PSYCHOLOGY-AND-PSYCHOTHERAPY-THEORY-RESEARCH-AND-PRACTICE OR PSYCHOPATHOLOGY OR PSYCHOPHARMAKOTHERAPIE OR PSYCHOSIS-PSYCHOLOGICAL-SOCIAL-AND-INTEGRATIVE-APPROACHES OR PSYCHOSOMATICS OR PSYCHOTHERAPY-AND-PSYCHOSOMATICS OR REVISTA-BRASILEIRA-DE-PSIQUIATRIA OR REVISTA-DE-PSIQUIATRIA-CLINICA OR REVISTA-DE-PSIQUIATRIA-Y-SALUD-MENTAL OR RIVISTA-DI-PSICHIATRIA OR SANTE-MENTALE-AU-QUEBEC OR SCANDINAVIAN-JOURNAL-OF-CHILD-AND-ADOLESCENT-PSYCHIATRY-AND-PSYCHOLOGY OR SCHIZOPHRENIA-BULLETIN OR SCHIZOPHRENIA-RESEARCH OR SOCIAL-ANXIETY-IN-CHILDHOOD-BRIDGING-DEVELOPMENTAL-AND-CLINICAL-PERSPECTIVES OR SOCIAL-PSYCHIATRY-AND-PSYCHIATRIC-EPIDEMIOLOGY OR SOCIAL-WORK-IN-MENTAL-HEALTH OR SOUTH-AFRICAN-JOURNAL-OF-PSYCHIATRY OR SUICIDE-AND-LIFE-THREATENING-BEHAVIOR OR SUICIDOLOGY OR SUICIDOLOGY-ONLINE-SOL OR THERAPEUTIC-ADVANCES-IN-PSYCHOPHARMACOLOGY OR TRANSLATIONAL-NEUROSCIENCE-IN-PSYCHIATRY OR TRANSLATIONAL-PSYCHIATRY OR TURK-PSIKIYATRI-DERGISI OR WORLD-JOURNAL-OF-BIOLOGICAL-PSYCHIATRY OR WORLD-JOURNAL-OF-PSYCHIATRY OR WORLD-PSYCHIATRY OR ZEITSCHRIFT-FUR-KINDER-UND-JUGENDPSYCHIATRIE-UND-PSYCHOTHERAPIE OR ZEITSCHRIFT-FUR-PSYCHIATRIE-PSYCHOLOGIE-UND-PSYCHOTHERAPIE)

TI=(12-STEP OR ADHD OR ADDICT* OR ADJUSTMENT-DISORDER* OR AFFECTIVE OR AGORAPHOBI* OR AKATHISIA OR ALEXITHYMIA OR ALPRAZOLAM OR ALZHEIMER* OR AMITRYPTILINE OR AMYLOID-BETA OR ANGER-MANAGEMENT OR ANORECTIC OR ANOREXI* OR ANTIDEPRESSANT* OR ANTIPSYCHOTIC* OR ANTISOCIAL OR ANXIETY OR ANXIOLYTIC* OR ANXIOUS OR APNEA OR APNOEA OR ARIPIPRASOLE OR ATTENTION-DEFICIT OR BEHAVIOR*-DISORDER* OR BEHAVIOR*-PROBLEM OR BEHAVIOR-THERAPY OR BETA-AMYLOID OR (BIPOLAR NOT (CELL-DIFFERENT* OR CLAMPING OR KNOT* OR LOCALIZ* OR OUTFLOW OR PATTERN OR SPINDLE OR TRANSISTORS)) OR BINGE-EAT* OR BINGEING OR BODY-IMAGE OR BORDERLINE-PERSONAL* OR BULIMI* OR BUPROPION OR ((ALCOHOL* OR AMPHETAMINE* OR BETEL OR CANNABI* OR COCAINE OR DRUG OR DRUGS OR ECSTASY OR HEROIN OR MARIJUANA OR MDMA OR METHAMPHETAMINE OR OPIATE* OR OPIOID* OR SUBSTANCE) AND (ABSTAIN* OR ABUSE OR ABUSING OR ADDICT* OR ADOLESCEN* OR CONSUMPTION OR CRAV* OR DEPENDEN* OR DRINK* OR EXPOSURE OR MEMORY OR MEN OR MICE OR MISUSE* OR PROBLEM* OR RAT OR RATS OR SELF-ADMINIST* OR (TREATMENT NOT (DRUG* OR HEAT)) OR (USE NOT FUEL) OR USER* OR VIOLEN* OR WITHDRAW* OR WOMEN)) OR CAPGRAS OR CARBAMAZEPINE OR CATATONI* OR CBT OR CHLORPROMAZINE OR CITALOPRAM* OR CLIOQUINOL OR CLOMIPRAMINE OR CLOZAPINE* OR COGNITIVE-BEHAVIOR* OR COGNITIVE-IMPAIR* OR COGNITIVE-THERAPY OR CONVERSION-DISORDER* OR DELIBERATE-SELF OR DELUSION* OR DEMENT* OR DEPERSONALIZATION OR DEPRESSED-PATIENT* OR (DEPRESSION NOT (MAGNETIC OR MOUSE OR RAT)) OR DEPRESSIVE* OR DESIPRAMINE OR DETOXIFICAT* OR DISSOCIATIVE-DISORDER OR DONEPEZIL OR DOTHIEPIN OR DULOXETINE OR DYSPHORI* OR DYSTHYMI* OR EATING-ATTITUDE* OR EATING-DISORDER* OR ELECTROCONVULSIVE-THERAP* OR EMDR OR EMOTIONAL OR ESCITALOPRAM OR FALSE-MEMORY OR FAMILY-THERAPY OR FLUANXOL OR FLUOXETINE OR FLUPENTIXOL OR FLUPHENAZINE OR FLUVOXAMINE OR FRIGIDITY OR FUNCTIONAL-SOMATIC-SYMPTOMS OR GABAPENTIN OR GALANTAMINE OR GAMBLING OR GARDENING-THERAPY OR GENDER-DYSPHORIA OR GRIEF-DISORDER OR GRIEF-THERAPY OR HALLUCINAT* OR HALLUCINOSIS OR HALOPERIDOL OR HYPERACTIVITY-DISORDER* OR HYPERSOMNIA OR HYPOMANIA OR HYSTERIA OR HYSTERICAL OR IMIPRAMINE OR IMPOTENCE OR INJECT-DRUGS OR INSOMNIA* OR IRRATIONAL-BELIEFS OR ISOCARBOXAZIDE OR LEARNING-DIFFICULT* OR LEARNING-DISABILIT* OR LOFEXIDINE OR LONELINESS OR MALOXANE OR MANIA OR (MANIC NOT FRINGE) OR MAPROTILINE OR MELANCHOLI* OR MEMANTINE OR MENTAL*-DISEASE* OR MENTAL*-DISORDER* OR MENTAL-HEALTH OR MENTAL-ILL-HEALTH OR MENTAL-HOSPITAL* OR MENTAL-ILLNESS OR MENTALLY-ILL OR METHADONE OR METHYLPHENIDATE OR MIANSERIN OR MINDFULNESS OR MIRTAZAPINE OR MOCLOBEMIDE OR MODAFINIL OR MOOD OR NALTREXONE OR NARCOLEPSY OR NARCISSISM OR NEEDLE-EXCHANGE OR NEUROLEPTIC* OR NEUROPSYCHIATR* OR NEUROSES OR NEUROSIS OR NEUROTIC* OR NORTRIPTYLINE OR OBSESSI* OR OCD OR OLANZAPINE OR PAEDOPHIL* OR PANIC OR PARANOID OR PARAPHILIA* OR PARAPHRENIA OR PARASUICID* OR PAROXETINE OR PEDOPHIL* OR PERSONALITY-DISORDER OR PHENELZINE OR PHOBI* OR PIMOZIDE OR PLAY-THERAPY OR POST-TRAUMATIC-STRESS OR POSTTRAUMATIC OR PRESENILIN OR PSYCHIATR* OR PSYCHOANALY* OR PSYCHODYNAMIC OR PSYCHOPATH* OR PSYCHOPHARM* OR PSYCHOS*S OR PSYCHOTIC OR PSYCHOGE* OR PSYCHOLOGICAL-STRESS OR PSYCHOMOTOR-AGITATION OR PSYCHOSOCIAL-REHABILITATION OR PSYCHOSOMATIC* OR PSYCHOTHERAP* OR PSYCHOTROPIC* OR PSYCHOTRAUMA* OR PTSD OR QUETIAPINE OR REBT OR RECOVERED-MEMORY OR REMOXIPRIDE OR RESTLESS-LEG OR RETT OR RETT*-SYNDROME OR RISPERIDONE OR RIVASTIGMINE OR ROBOXETINE OR ROSIGLITAZINE OR SCHIZOAFFECTIVE OR SCHIZOID OR SCHIZOPHRENI* OR SCHIZOTYP* OR SELECTIVE-SEROTONIN-REUPTAKE OR SELF-ACCEPTANCE OR SELF-CUTTING OR SELF-ESTEEM OR SELF-HARM* OR SELF-INJUR* OR SELF-MUTILAT* OR SELF-POISON* OR SERTRALINE OR SEXUAL-DYSFUNCTION OR SOMATIZ* OR SOMATOFORM OR SOMNOLENCE OR SSRI OR SSRIS OR (SUICID* NOT (CELL* OR PLASMID* OR RAT)) OR TARDIVE-DYSKINESI* OR THIORIDAZINE OR TOPIRAMATE OR TOURETTE* OR TRANSSEXUAL OR TRANYLCYPROMINE OR TRAZODONE OR TRICHOTILLOMANIA OR TRIFLUOPERAZINE OR TRIMIPRAMINE OR VALPROATE OR VENLAFAXINE OR WELL-BEING OR ZIPRASIDRONE)

(#1 OR #2) NOT ((WC=(AGRICULTUR* OR AGRONOMY OR ARCHAEOLOGY OR ARCHITECTURE OR AREA STUDIES OR ART OR ASIAN STUDIES OR ASTRONOMY & ASTROPHYSICS OR AUTOMATION & CONTROL SYSTEMS OR BIODIVERSITY CONSERVATION OR BIOLOGY OR BIOPHYSICS OR BUSINESS* OR CLASSICS OR COMMUNICATION OR CONSTRUCTION & BUILDING TECHNOLOGY OR CRYSTALLOGRAPHY OR CULTURAL STUDIES OR DANCE OR DEMOGRAPHY OR DENTISTRY* OR DERMATOLOGY OR DEVELOPMENTAL BIOLOGY OR ECOLOGY OR EDUCATION* OR ENERGY & FUELS OR ENGINEERING* OR ENTOMOLOGY OR ENVIRONMENTAL* OR ERGONOMICS OR ETHICS OR ETHNIC STUDIES OR EVOLUTIONARY BIOLOGY OR FILM* OR FISHERIES OR FOLKLORE OR FOOD SCIENCE & TECHNOLOGY OR FORESTRY OR GEO* OR GREEN* OR HISTORY* OR HORTICULTURE OR HOSPITALITY* OR HUMANITIES* OR INDUSTRIAL RELATIONS & LABOR OR INFORMATION SCIENCE & LIBRARY SCIENCE OR INSTRUMENTS & INSTRUMENTATION OR INTERNATIONAL RELATIONS OR LANGUAGE & LINGUISTICS OR LAW OR LIMNOLOGY OR LINGUISTICS OR LITERA* OR LOGIC OR MANAGEMENT OR MARINE & FRESHWATER BIOLOGY OR MATERIALS* OR MECHANICS OR MEDIEVAL & RENAISSANCE STUDIES OR METALLURGY & METALLURGICAL ENGINEERING OR METEOROLOGY & ATMOSPHERIC SCIENCES OR MICROSCOPY OR MINERALOGY OR MINING & MINERAL PROCESSING OR MUSIC OR MYCOLOGY OR NANOSCIENCE & NANOTECHNOLOGY OR OCEANOGRAPHY OR OPERATIONS RESEARCH & MANAGEMENT SCIENCE OR ORNITHOLOGY OR PALEONTOLOGY OR PHILOSOPHY OR PHYSICS* OR PLANNING & DEVELOPMENT OR PLANT SCIENCES OR POETRY OR POLITICAL SCIENCE OR POLYMER SCIENCE OR RELIGION OR REMOTE SENSING OR REPRODUCTIVE BIOLOGY OR ROBOTICS OR SOIL SCIENCE OR SPECTROSCOPY OR TELECOMMUNICATIONS OR THEATER OR THERMODYNAMICS OR TRANSPORTATION* OR URBAN STUDIES OR WATER RESOURCES OR ZOOLOGY)) OR (TI=(CAFFEINE OR (CANCER AND RISK) OR CIGARETTE OR FUEL OR NICOTINE OR PAIN OR SMOKER OR SMOKING OR TOBACCO) OR (WC=CHEMISTRY* AND TI=ETHANOL)))
